# The Sterilisation of Under-Clothing

**Published:** 1902-12-20

**Authors:** 


### THE STERILISATION OF UNDER-CLOTHING.

In a recent number of *Lyons Medical* attention is drawn to the frequency of cutaneous eruptions among the children at certain crèches. These eruptions took the form of boils, pustules, and erythematous rashes, and for several years had been very prevalent at the Crèche de la Charité. The use of antiseptics in the form of lotions and ointments had failed to stay the outbreak. The plan was then tried of using sterilised cloth for dressing the children, and at once an improvement in their condition manifested itself. The infection ceased to spread to new cases except on portions of the scalp, and the use of sterilised caps soon put a stop to this, while, so far as the children already affected were concerned, they began to improve from the day on which the treatment was commenced. The matter is of some interest, and one can hardly avoid asking what is meant by the sterilisation of under-linen? Surely it must mean that it is boiled. It used to be, and perhaps still is, the custom in France to wash clothes in cold running water, a plan by which, however thoroughly they may be lathered and rubbed and beaten, they certainly are not sterilised.

Years ago a virulent outbreak of puerperal fever in one of the London lying-in hospitals was traced to the manner in which the linen of the establishment was dealt with. It was washed, undoubtedly, but it was not boiled, and on its return, in a more or less damp condition, it was stored in a warm closet wherein it was imagined to be "aired." The result was that the cupboard acted as an incubator in which the infection remaining in this damp linen flourished exceedingly, with results which were disastrous. In this case, as probably in the case reported from Lyons, we had an instance of the evil consequences of not thoroughly sterilising the underclothing. The moral is sufficiently obvious. How then about woollen materials which, lest they should shrink, are merely washed in tepid water? If this were done at home there might not, perhaps, be much to say on the subject, but how about mixing woollen underclothes, fresh from the skins of scores of unknown people, in one common tub, and then, without any pretence at sterilisation, returning them to their respective owners? That woollen under-wear is comfortable, and that in certain cases it affords a degree of protection from chill which cannot be obtained from any other form of clothing we fully admit. We must not, however, shut our eyes to the risks involved in the co-operative washing of clothes, the texture of which does not admit of their being sterilised by exposure to a high temperature.

